# Recommendations for a Combined Laparoscopic and Transanal Approach in Treating Deep Endometriosis of the Lower Rectum—The Rouen Technique

**DOI:** 10.3390/jpm11050408

**Published:** 2021-05-13

**Authors:** Şerban Nastasia, Anca Angela Simionescu, Jean Jacques Tuech, Horace Roman

**Affiliations:** 1 Department of Obstetrics and Gynecology, Carol Davila University of Medicine and Pharmacy, 050474 Bucharest, Romania; serban_nastasia@yahoo.com; 2 Department of Obstetrics and Gynecology, Filantropia Clinical Hospital, Carol Davila University of Medicine and Pharmacy, 11–13 Ion Mihalache Blv, District 1, 011171 Bucharest, Romania; 3 Digestive Tract Research Group EA3234/IFRMP23, Department of Digestive Surgery, Rouen University Hospital, 76031 Rouen, France; jean-jacques.tuech@chu-rouen.fr; 4 Centre d’endométriose, Clinique Tivoli-Ducos, 91 rue Rivière, 33000 Bordeaux, France; horace.roman@gmail.com

**Keywords:** low rectovaginal deep endometriosis, surgical education, Rouen technique, laparoscopic-transanal disc excision, plasma energy shaving

## Abstract

The complete excision of low rectovaginal deep endometriosis is a demanding surgery associated with an increased risk of intra- and postoperative complications, which can impact the quality of life. Given the choices of optimal surgery procedures available, we would like to emphasize that a minimally invasive approach with plasma medicine and a transanal disc excision could significantly improve surgery for deep endometriosis, avoiding the lateral thermal damage of vascular and parasympathetic fibers of roots S2–S5 in the pelvic plexus. The management of low rectal deep endometriosis is distinct from other gastrointestinal-tract endometriosis nodules. Suggestions and explanations are presented for this minimal approach. These contribute to individualized medical care for deep endometriosis. In brief, a laparoscopic transanal disc excision (LTADE; Rouen technique) was performed through a laparoscopic deep rectal dissection, combined with plasma energy shaving, and followed by a transanal disc excision of the low and mid-rectal deep endometriotic nodules, with the use of a semi-circular stapler. LTADE is indicated as the first-line surgical treatment for low and mid-rectal deep endometriotic nodule excisions, because it can preserve rectal length and innervation. This technique requires a multidisciplinary team with surgical colorectal training.

## 1. Introduction

Deep endometriosis of the rectum involves the muscular layer of the rectal wall [1] with a depth of more than 5 mm [2,3], and choosing an optimal surgical treatment is challenging. Endometriosis is considered a multifactorial [4], multifocal disease with genetic predisposition [5] and a long-term unpredictable evolution. Deep infiltrating endometriosis of the rectum is a chronic and evolutive disease with a high capacity for aggressive cells. Deep infiltrating endometriosis of the rectum leads to severe symptoms, such as progressive pelvic pain associated with dysmenorrhea, deep dyspareunia, and various digestive complaints such as diarrhea, constipation, tenesmus, dyschezia, painful defecation, and occlusion. Patients experience a significant impairment of professional and social lives. The rectum and rectosigmoid junction account for up to 90% of endometriotic intestinal lesions, making them difficult to manage [6,7]. Several surgical techniques have been used for deep symptomatic bowel and rectovaginal endometriosis, including colorectal resection, disc excision, and shaving combined with the excision of the rectovaginal endometriosis nodule [3,8]. Each of these techniques has shown efficacy in treating deep endometriosis. After surgery, different recurrence rates were reported, but recurrence and repeated surgery were not always a consequence of an incomplete endometriosis resection [9,10]. When deep symptomatic endometriosis involves the middle and lower rectum, a low colorectal resection is thought to eradicate endometriosis. The management of low rectal deep endometriosis is distinct from other gastrointestinal tract endometriosis nodules. Low colorectal resection may be followed by unfavorable functional outcomes, such as the low anterior rectal resection syndrome [11,12], mainly due to somatic and autonomic pelvic nerve damage. Disc excision and shaving combined with the excision of the rectovaginal endometriosis nodule have produced better clinical post-operative outcomes, such as urinary retention, rectovaginal fistula, post-operative rectal stenosis, and pregnancy [8,13,14]. Immunopathological, molecular, and genetic differences between asymptomatic and symptomatic cases at the level of endometriosis implants, eutopic endometrium, and the peritoneal environment were accordingly identified with the intracellular production of estrogens [15,16,17].

Given that optimal surgery procedure choice needs to be individualized, we want to emphasize that a minimally invasive approach with plasma medicine could significantly improve surgery for deep endometriosis, avoiding the lateral thermal damage of vascular and parasympathetic fibers of roots S2–S5 from the pelvic plexus.

The Rouen technique (laparoscopic-transanal disc excision (LTADE)) was conceived (by RH and TJJ) to avoid a segmental resection of the low rectum and to reduce the risk of rectal stenosis and denervation [18,19,20].

This minimal approach combines laparoscopic deep rectal dissection and shaving with plasma energy, followed by a transanal disc excision of mid-rectal deep endometriotic nodules with a semi-circular stapler (Contour Transtar stapler, Ethicon EndoSurgery Inc., Cincinnati, OH, USA). Last decade, technical adjustments and short and lifelong outcomes of this surgery approach were described in various published reports. None of the reports addresses all the possible technical challenges.

In this study, we aimed to combine all the available published information in one article that constructs a comprehensive but straightforward and pragmatic approach when performing a disc excision of large nodules of deep endometriosis infiltrating the lower and middle rectum with the Rouen technique. Rectal shaving using the PlasmaJet System has the advantage of a targeted ablation of rectal endometriotic nodules, the lack of lateral thermal spread around the plasma jet due to high kinetic energy and the use of highly controlled thermal effects. The PlasmaJet System enhances the anatomic and atraumatic dissection of the subperitoneal space. This approach may raise awareness of the operative experience for specialists that perform surgery for endometriosis.

## 2. Surgical Procedure

This technique is suitable for removing nodules located low in the rectum (5.5 ± 1.3 cm from the anal verge), particularly when the posterior vaginal wall is involved (which occurs in up to 83.3% of the cases) [21]. Surgery was indicated after careful clinical evaluation using preoperative quantitative questionnaires on gynecological, digestive, and general symptoms [22,23,24,25], as well as an intrarectal ultrasound, an MRI, and a computed tomography-based virtual colonoscopy examination. Deep endometriosis in the recto-vaginal septum generates anatomic and functional modifications due to fibrosis and endometrial infiltration, followed by the adherence of the recto-sigmoid to the lower dorsal side of the uterus, cervix, and vagina. Recto-vaginal septum contains nerve structures from the inferior hypogastric plexus, uterovaginal plexus, vesical nerve, and the lower rectal plexus, while multiple branches and anastomoses of the inferior mesenteric artery or internal iliac artery play a role in continence, defecation, and in sexuality [3,26].

**Figure 1:**
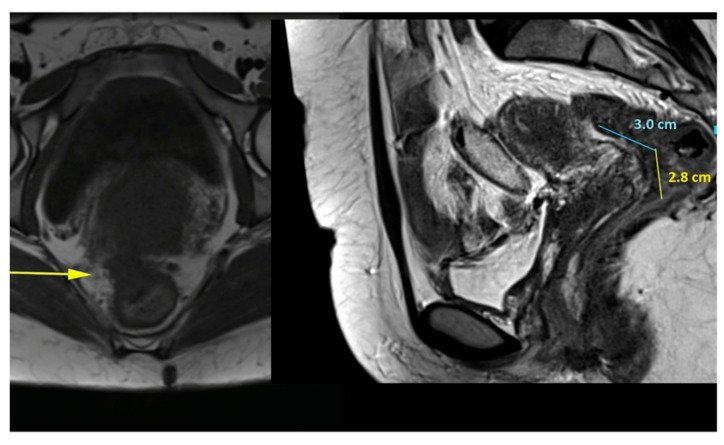
Preoperative MRI image shows deep endometriosis involving the low rectum and vagina.

The patient acknowledged and signed the Informed Consent for the treatment and for the use of this case for educational and scientific research purposes. In Figure 1 we present a Preoperative MRI image that shows deep endometriosis involving the low rectum and vagina.

**Figure 2:**
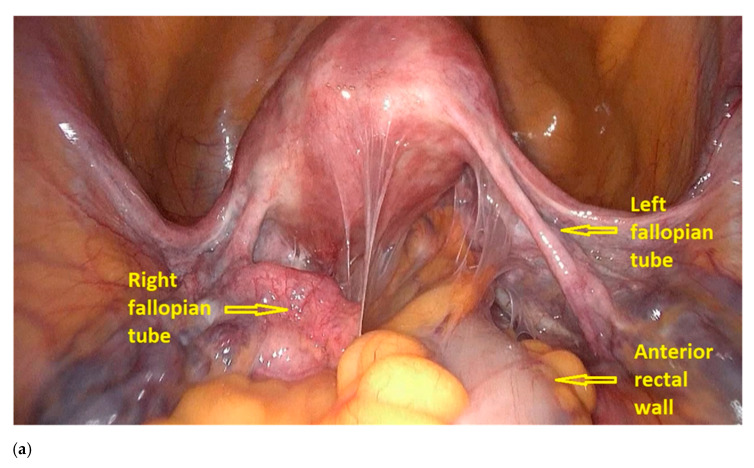

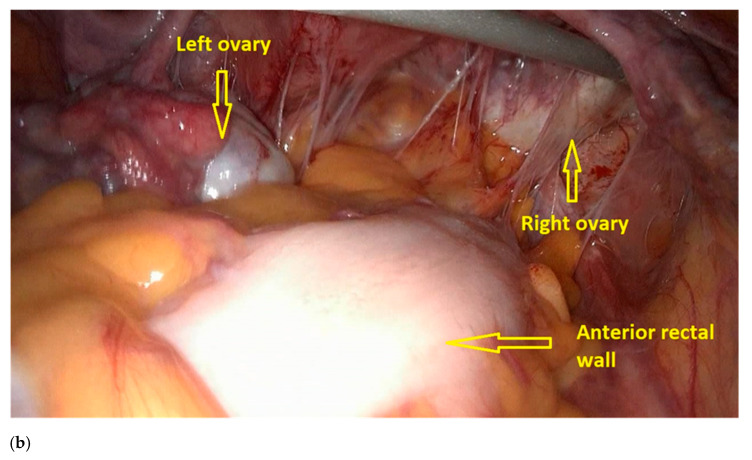
Laparoscopic view of the pelvis. (**a**) Inspection of the pelvic cavity and (**b**) identification of anterior rectal wall.

**Figure 3:**
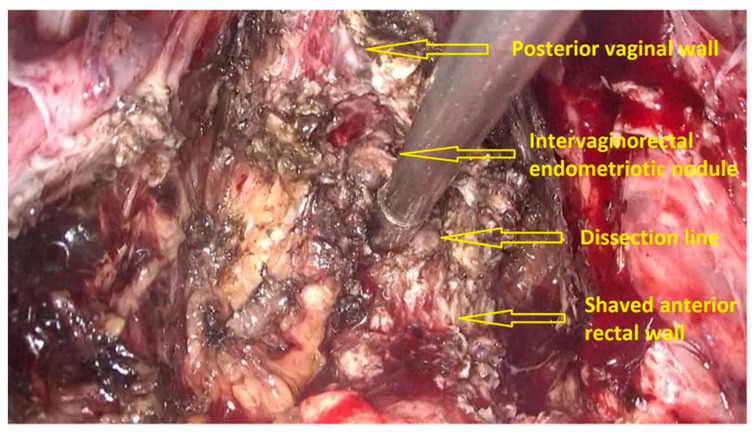
Opening the deep rectal spaces and rectovaginal septum surrounding the rectal nodule. The nodule is dissected, and the rectum is released and shaved.

**Figure 4:**
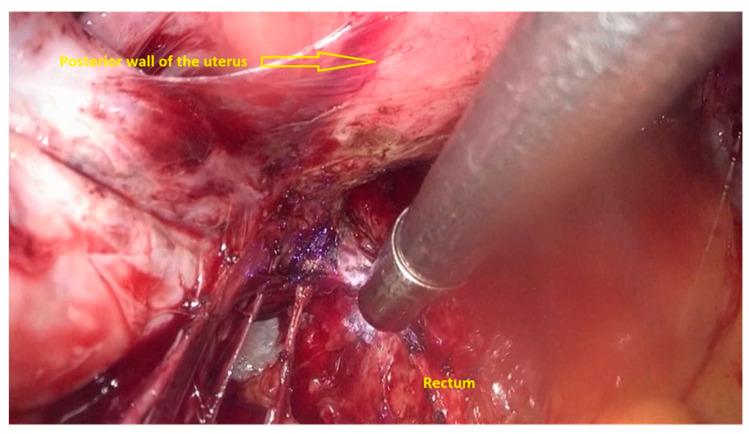
Dissection and removal of the fat tissue on the left lateral rectal wall.

**Figure 5:**
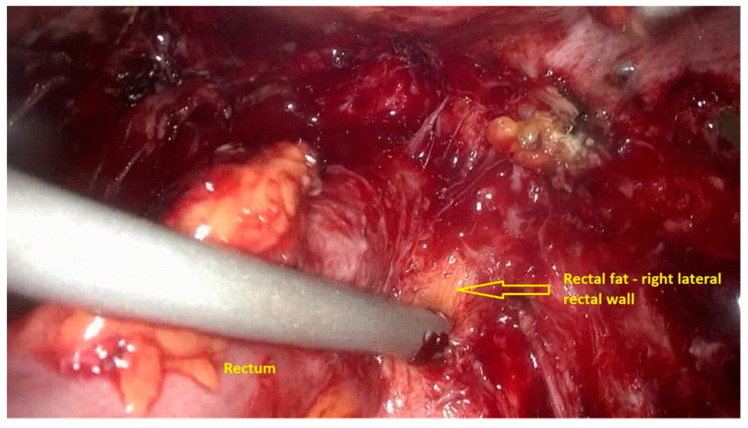
Dissection and removal of the fat tissue on the right rectal wall.

**Figure 6:**
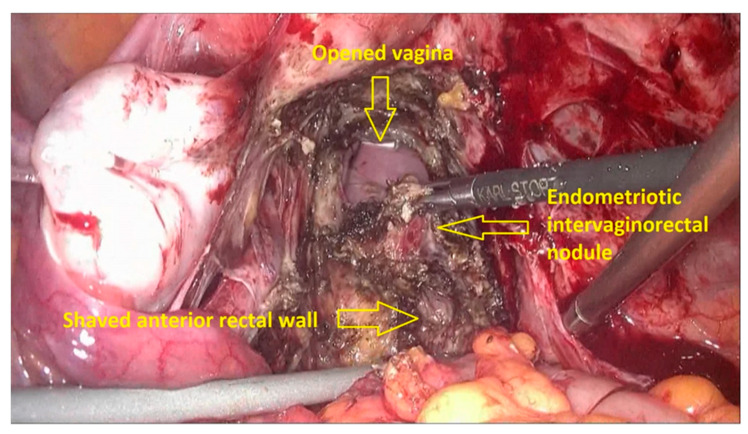
Excision of a vaginal patch, followed by vaginal closure.

The first step of the procedure is performed laparoscopically. The anterior rectum is dissected free from the posterior vagina, with the separation of the rectovaginal nodule of endometriosis. Deep rectal shaving is performed with plasma energy. This step proceeds as follows:

- The procedure starts with the inspection of the pelvic cavity and identification of the anterior rectal wall (Figure 2a,b).
- The deep rectal spaces and rectovaginal septum surrounding the rectal nodule are opened in an anterolateral plan while staying connected to the levator ani muscle and with the preservation of fascia recti (Figure 3).
- This dissection is followed by the removal of fat tissue on the lateral rectal walls (Figure 4 and Figure 5) with the preservation of pre-sacral fascia.
- After shaving, the rectum is completely freed. However, the shaved area might be rigid and infiltrated by endometriotic foci. When present, vaginal infiltration requires the excision of a patch of the posterior vaginal wall (Figure 6).

**Figure 7:**
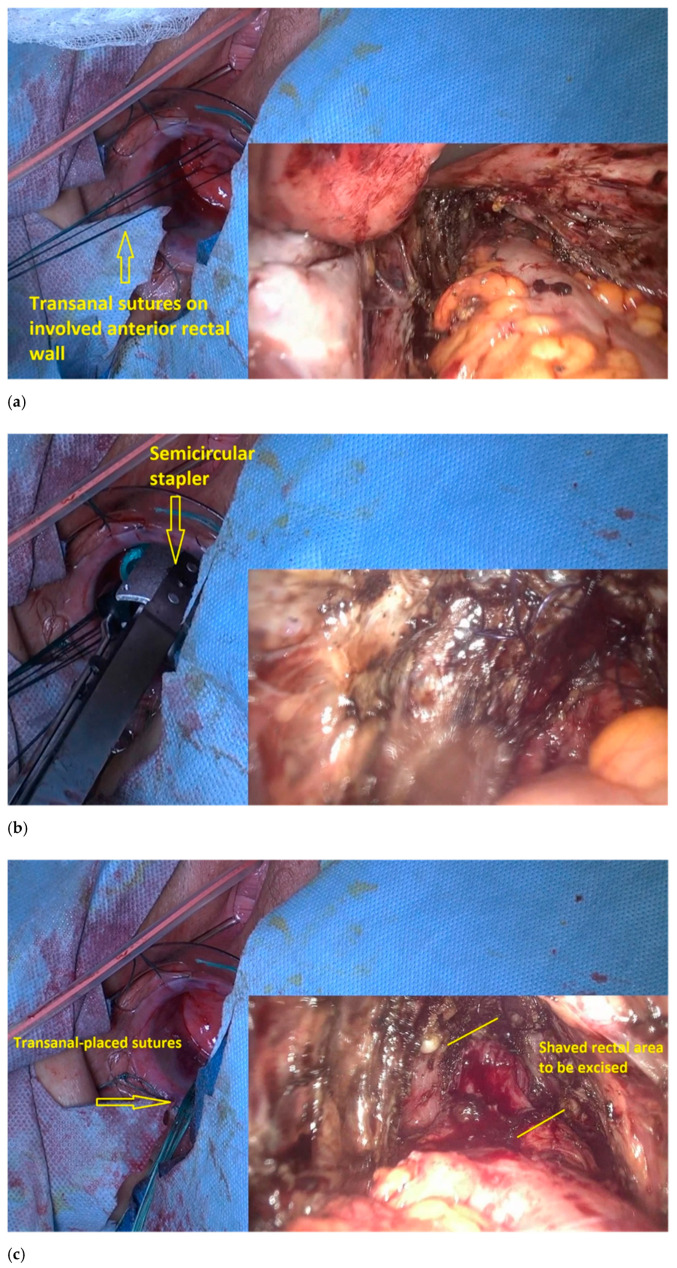

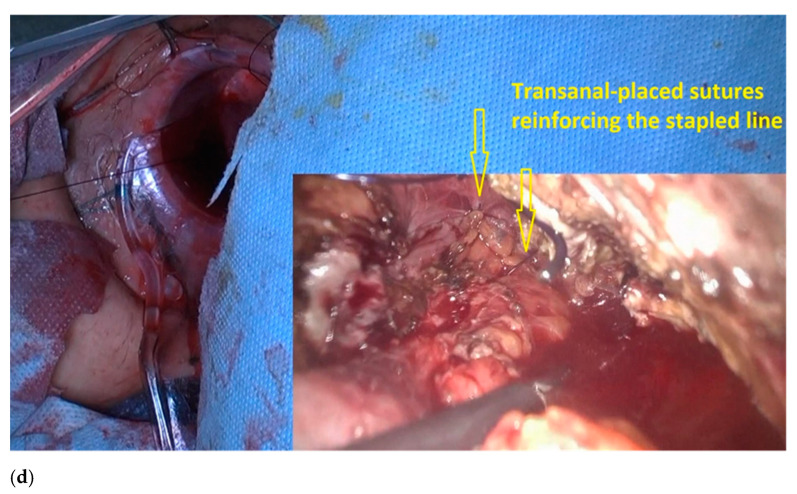
Transanal excision of the involved rectal area. (**a**) Transanal placement of a suture on the shaved area; (**b**) laparoscopic placement of a suture on the shaved area, to assist the colorectal surgeon in identifying the rectal area to be excised; (**c**) introduction of the closed transanal circular stapler (the stapler opening is at the nodule level), and the stapler closing and firing; (**d**) stitches reinforce the stapled line.

The second step of the procedure is performed using the transanal approach. This step involves the excision of low rectal infiltration (Figure 7).

- The transanal dilatator is introduced to identify the shaved area. Once the shaved area is identified, with simultaneous transanal and laparoscopic views, 3 or 4 traction parachute sutures are placed in the middle and outside the shaved area (Figure 7a). The gynecologic surgeon uses laparoscopy to check the correct placement of the stitches and makes sure that the vagina is not caught in the stitching.
- The traction of the stitches induces the prolapse of the shaved rectum wall into the rectal lumen, which facilitates resection with the semicircular stapler, a device designed initially for excising a rectal prolapse (Figure 7b).
- A laparoscopically placed suture over the shaved rectal area assists the colorectal surgeon in correctly identifying the area to be resected (Figure 7c).
- The lubricated head of the stapler is introduced at the 3 o’clock position, with the jaws facing counterclockwise. The device is then rotated, and the shaved area is gently pulled inside the jaws until the surrounding normal bowel wall is set within the jaws.
- The stapler retaining pin is then applied, and the stapler is closed around the tissue for 15 s to maximize tissue compression; the staple is subsequently fired, and then removed. The stapler cartridge is then replaced, and the device is reintroduced into the rectum. This procedure is repeated until the shaved rectal area is completely resected. The stapled lines are inspected for bleeding. Reinforcement sutures are placed transanally, when necessary (Figure 7d).

- An air test is performed to ensure the integrity of the stapled line.
- A generous omentum flap is placed between the rectal and vaginal suture sites.
- When the procedure is associated with a large vaginal resection and a large low rectal excision, a diverting stoma on the sigmoid colon may be performed, based on the risk of developing fistula or anastomotic leakage.

A video demonstrating the proper technique is available as a Supplementary Material to this paper.

Before 2020, this technique was performed in 88 cases, with one rectal recurrence (1.1%), and a leakage rate of 10.2% (unpublished data). The mean surgery time was 162 ± 72 min. The mean diameter of rectal specimens was 57 ± 10 mm [20]. Klapczynski et al. show that the procedure resulted in long-term satisfaction, including the decrease in the occurrence of the low anterior resection syndrome (LARS), as well as functional outcomes and successful pregnancies. A total of 15% of patients presented major rectal disfunction, and 62.5% had a normal postoperative rectal function. Moreover, patient satisfaction evaluations showed satisfactory scores. The development of a rectovaginal fistula was not corelated with the risk of major rectal disfunction (adjusted OR 6, 3, 95% CI 1, 3–30, 6) [27].

## 3. Discussion

The Rouen technique facilitates the precise and complete excision of large macroscopic nodules involving deep endometriosis in the mid and low rectum, particularly with the involvement of the posterior vaginal wall. This is due to the specific properties of PlasmaJet—the targeted and precise ablation of the rectal endometriosis implants and the absence of lateral thermal spread around the plasma jet make the dissection on the rectal wall safe, as well as the dissection of subperitoneal spaces using enhanced Plasma Jet kinetic energy [19,28]. The surgical approach showed a favorable post-operative evolution in gynecologic and digestive functions including pelvic pain relief symptoms [29]. Clinical trials reported a significantly lower rate of severe neurologic pelvic dysfunctions associated with severe bladder/rectal/sexual dysfunctions when nerve-sparing surgery was performed [30]. Bowel occult microscopic endometriotic lesions after colorectal resection had no impact on short-term postoperative outcomes.

Compared to segmental colorectal resection, the transanal disc excision on the anterior rectal wall in rectal endometriosis in the Rouen technique can preserve the mesorectum, spare the rectal vessels and nerves, and preserve the length and capacity of the rectal ampulla [21]. Nerve damage can negatively impact postoperative rectal function [31]. Nerve-sparing techniques have also been recommended for preserving the inferior hypogastric plexus, hypogastric nerves, and splanchnic nerves, at least on one side. There are arguments that performing disk excision instead of low rectal resection significantly increases the probability of postoperative normal bowel movements.

However, nerve damage may not be avoided with surgery due to endometriosis infiltration, particularly in cases that involve large, deep nodules in the parametrium.

The preservation of the rectum may not be the major concern in preserving rectal function, particularly when the parametrium and nerves are infiltrated with endometriosis. The rectal shape may be conserved, although it may no longer be innervated accurately. Furthermore, studies by Mabrouk et al. [32] have shown that patients with colorectal endometriosis may have preoperative anal and urethral sphincter hypertonia. This condition can cause rectal or bladder dysfunction, which cannot be restored by removing nodules.

However, the Rouen technique has been associated with a high percentage of rectovaginal fistulae due to the large size of low rectal and parametrium-located endometriosis nodules. Deep endometriosis nodules infiltrating the low rectum usually involve the adjacent vagina, resulting in a concomitant rectal and vaginal excision, which may increase the risk of rectal fistula and an anastomotic leakage, and its subsequent complications. After low rectum segmental resection, the use of preventive stoma (Ref.) [33] may be followed by rectal dysfunction due to a higher postoperative risk of stenosis of the colorectal anastomosis [34]. This complication has not been reported after disk excision [34]. Performing routine stoma does not preclude the risk of rectovaginal fistula. A rectal stapled line at a height of <8 cm was an independent major risk factor for rectovaginal fistula, irrespective of preventive stoma usage. A valuable alternative to the stoma is the ghost ileostomy, a safe and effective alternative to routine ileostomy that is associated with a 10.5% rate of exteriorization and a 2.1% rate of adverse events [35].

A retrospective multicentric study published recently by Bokor et al. that compared the Rouen technique to a nerve and vessel-sparing segmental resection found a significantly higher rate of rectovaginal fistulae in the Rouen disc excision arm of the study (10.6% versus 3.6%) [36]. A total of 139 women enrolled at three European university hospitals, who had undergone segmental rectal resection with colorectal anastomosis below 7 cm from the anal verge, were compared to 66 women who underwent surgery using the Rouen technique. The 66 women enrolled in the disk excision group were diagnosed with large and low localization of the rectal nodules. However, it should be noted that the reported 3.6% rate of rectovaginal fistulae after colorectal resection include all localizations and all sizes of deep colonic endometriosis. The rate of rectovaginal fistulae after colorectal resection for low rectal deep endometriosis was more than 10%. The vaginal excision, particularly when more than 3 cm of the vagina is involved, results in the approximation of vaginal and low rectum sutures, which further increase the risk of rectovaginal fistulae.

The reported rates of rectovaginal fistulae from systematic reviews were [37] found at 2.7% after colorectal resection, with the rate of anastomosis leakage at 1.5%. However, in several retrospective series reported by experienced surgeons who routinely perform bowel resection in deep endometriosis, the rate of rectovaginal fistulae rose to 8.4% [38] or 10.7% [39], whereas that of anastomotic leakage rate was at 2.1% [40] or 4.7% [41].

The Rouen technique can remove large nodules of up to 7 cm in diameter, though their superior limit should not exceed 8–9 cm, to allow for mobilization down to the anal verge [42]. As a consequence, patients managed with the Rouen technique usually present a more severe disease involving not only the low rectum but also the vagina and the parametrium, which may theoretically further increase the risks of rectovaginal fistula or bladder dysfunction. However, the risk of fistulas is counterbalanced by the decrease in the LARS [36].

The LARS following a rectal resection includes short and long-term symptoms: urgency, constipation, feeling of incomplete emptying, clustering of stools, and frequency. An evaluation conducted at least 6 months postoperatively showed that, among women managed with the Rouen technique, 85% showed normal or low scores [33]. Moreover, a large anterior disc excision of the low and mid rectum anterior wall spared the posterior rectal wall length. Postoperative MRI studies of the rectum shape revealed a posterior rectal pouch in all patients that received the Rouen technique; however, none of those patients presented with rectal stenosis [31,43]. Nonetheless, one year after the Rouen technique, functional digestive outcomes were not correlated with the size of the posterior pouch [43].

Endometriosis is a cause of infertility [44]. It is well-known that surgical treatment for ovarian endometriomas may determine a diminished ovarian reserve and poor ovarian response to stimulation. Fertility rate after colorectal resection was estimated at 46.9%, whereas that of spontaneous conception averaged 28.6% [45]. Spontaneous pregnancies were more frequent after the Rouen technique (more than 50%) than after laparoscopic segmental resection (39%) [44]. However, when patients with severe or moderate endometriosis are referred to fertility centers, primary in vitro fertilization (IVF) would automatically be offered. For these reasons, reporting postoperative pregnancy rate in patients managed for colorectal endometriosis is both difficult and meaningful. Along with painful complaints and postoperative improvement, spontaneous conception remains a major concern with regard to health expenses and patient comfort [21]. Another series on a small number of patients suggested that surgery improved both fertility and pregnancy rates after the Assisted Reproductive Technology (ART) [44,46].

## 4. Conclusions

The Rouen technique enables the excision of large nodules involving the low rectum and avoids the occurrence of the LARS. This technique is appropriate for removing nodules located low in the rectum, particularly when the posterior vaginal wall is involved. Surgical management of deep rectal endometriosis depends on the general characteristics of the patient (age, parity, symptoms), on their subsequent desire of pregnancy, the surgeon’s experience, and the types of equipment available. The choice of the most optimal surgical method needs to be personalized by the multidisciplinary team. Disc excision of large endometriosis nodules in the low rectum with the Rouen technique provided good functional outcomes. The strength of this surgery is its low risk for developing the low anterior resection syndrome, which is typically associated with distal rectum surgery.

## Supplementary Materials

The following are available online at https://www.mdpi.com/article/10.3390/jpm11050408/s1, Video S1: Tips and tricks for a combined laparoscopic and transanal approach for treating deep endometriosis of the lower rectum—the Rouen technique.
